# Promoting antimicrobial stewardship on dairy farms in Mekelle, Northern Ethiopia: a field intervention with One Health implications

**DOI:** 10.1186/s12917-026-05385-z

**Published:** 2026-03-09

**Authors:** Haftom Yirga Tsegay, Tadelech Yilma Sisay, Mebrahtu Weldegebriel Hailu, Werkey Araya Tekleargis, Muuz Gebru Sahle

**Affiliations:** 1 Department of Veterinary Basics and Diagnostic Sciences, College of Veterinary Sciences, Mekelle University, Mekelle, Ethiopia; 2 Department of Veterinary Clinical Medicine and Epidemiology, College of Veterinary Sciences, Mekelle University, Mekelle, Ethiopia; 3 Doctoral School of Biology and Environmental Sciences, University of Debrecen, Debrecen, Hungary

**Keywords:** Antimicrobial resistance, Antimicrobial stewardship, Antimicrobial use, Dairy farm, Disease incidence, Farmer training, One Health

## Abstract

**Background:**

Inappropriate antimicrobial use (AMU) in livestock contributes to antimicrobial resistance (AMR), posing risks to both animal and human health. Despite growing awareness, antimicrobial stewardship (AMS) initiatives remain limited in Ethiopia’s dairy sector. This study evaluated the effects of farm-level AMS training on farmers’ knowledge and attitudes toward AMR and AMU, and disease incidence on dairy farms in Mekelle, Northern Ethiopia.

**Methods:**

A prospective field intervention was conducted from December 2024 to June 2025 among 22 dairy farms with at least 20 lactating cows each. Farms were allocated to intervention (*n* = 11) and control (*n* = 11) groups based on geographical proximity. Baseline assessments preceded AMS training, with follow-up evaluations at three and six months. The AMS intervention included participatory training on prudent AMU, infection prevention, and record-keeping. Changes in farmers’ knowledge and attitudes were measured using structured questionnaires; AMU was quantified as treatments per 100 cow-days, and disease incidence as new cases per 100 cow-days.

**Results:**

Following AMS training, mean knowledge scores in the intervention group increased from 48.2% to 78.6% (*p* < 0.05), while control farms did not significantly change (47.3% to 51.5%). Attitude scores changed from 3.1 to 4.2 (*p* < 0.05). Antimicrobial treatment rates changed from 4.5 to 2.0 per 100 cow-days in the intervention group (56% reduction), with minimal change in controls (4.1 to 3.9). Disease incidence declined from 7.9 to 4.5 cases per 100 cow-days, a 43% reduction, with the largest decreases observed for mastitis (52%) and respiratory disease (47%).

**Conclusion:**

Targeted AMS training was associated with change of farmers’ knowledge and attitudes, reduction of AMU, and decreased disease incidence on dairy farms. These findings may show the effect of participatory, farm-level stewardship programs in promoting responsible AMU and herd health, with potential benefits for One Health. Sustained follow-up and integration into veterinary extension services are recommended to maintain long-term impact.

**Supplementary Information:**

The online version contains supplementary material available at 10.1186/s12917-026-05385-z.

## Background

Antimicrobials are important for treating infections of different types in dairy animals. The effectiveness of antimicrobials continues whenever they are properly used [1]. Antimicrobial resistance (AMR) refers to the state in which microorganisms cease to respond to antimicrobial medications. It is a naturally occurring process that occurs gradually as pathogens undergo genetic changes [2]. To slow the occurrence of AMR, appropriate use of antimicrobials is necessary [3].

Infectious diseases pose a significant threat to dairy production. They can cause illnesses in dairy animals, resulting in decreased milk production, lower milk quality, increased mortality rates, and additional costs for farmers. Mastitis, metritis, retained placenta and lameness are common diseases in dairy cattle [4, 5]. Traditionally, antibiotics have been used to treat these infections. However, the emergence of antimicrobial-resistant bacteria makes effectively treating these infections more challenging [6]. Microorganisms associated with diseases of dairy cattle, such as *Escherichia coli* and *Staphylococcus aureus* are the most studied causes in the study area, with alarming levels of AMR such as 90–100% resistance to penicillin-G [7, 8].

Antimicrobial use (AMU) in livestock production systems, particularly in dairy farms, has direct implications for the emergence and dissemination of AMR across the human-animal-environment interface [9–11]. Resistant bacteria and resistance genes originating from animal production can transfer to humans through direct contact, contaminated milk and meat, or environmental pathways such as manure and water runoff [10, 11]. Consequently, AMR in livestock production is not merely an animal health problem but a growing public health concern that requires coordinated action within a One Health framework [9].

Antimicrobial stewardship (AMS) is a coordinated program that promotes the appropriate use of antimicrobials to reduce AMR and decrease the spread of infections caused by drug-resistant organisms [12–14]. The main aim of AMS programs is to reduce the need for AMU by promoting its effective and appropriate use, as well as by focusing on infection control and prevention measures [14, 15]. Comprehensive education and training initiatives are essential components of successful AMS programs and help to make informed decisions in antimicrobial therapy [16, 17].

A prescription written by a veterinarian is required for most antimicrobial drugs used in livestock, with fewer drugs being available over the counter. However, farmers and dairy farm workers are most commonly the ones who diagnose diseases and make daily treatment decisions for animals on the farm [18, 19]. This shows the significance of farmworkers’ knowledge and skills related to disease diagnosis and therapy decision-making, as they directly affect the successful judicious use of antimicrobials in livestock production systems. Accurate identification of abnormal animal behavior and clinical signs of disease is crucial for clinical diagnostic skills and to improve treatment success.

AMS programs that provide training to farm workers have been shown to improve knowledge and skills in responsible AMU. Studies have shown that, by improving early identification of clinical signs of disease and implementing appropriate treatment protocols, farmworkers can effectively manage cattle sickness and improve treatment success [20, 21]. A study conducted by Seibt et al.. also demonstrated that farmworkers who receive AMS training show significant improvements in knowledge and attitudes towards AMU [22]. Similarly, Garzon et al.. reported measurable improvements in worker understanding and behavioral change following AMS education [21]. Other related research indicates that the effectiveness of AMS programs is enhanced when educational content is delivered through interactive, language-appropriate, and experience-sensitive methods [23, 24]. Together, these findings demonstrate that empowering farm workers through targeted and practical training is central to achieving responsible AMU and improving herd health outcomes within dairy production systems.

Beyond improving animal health and productivity, AMS programs in livestock farming have wider implications for global health [25–27]. Prudent AMU on farms directly contributes to slowing the spread of resistant bacteria that threaten treatment outcomes in humans [26, 27]. Integrating AMS education and surveillance into livestock systems thus represents a practical and locally implementable pathway for advancing the global One Health agenda [25].

No study focusing on AMS programs on dairy farms has been conducted in the study area. Therefore, this is the first study to examine AMS program on dairy farms in Mekelle and in the Tigray region of Ethiopia to evaluate the effects of an AMS training program on farm workers’ knowledge and attitudes regarding AMR and AMU, the quantity of antimicrobial treatments administered, and the incidence rate of common infectious diseases in dairy cattle; which has a great significance in the One Health framework.

## Methods

### Study design, study setting and period

A longitudinal quasi experimental study with parallel intervention and control groups was conducted on dairy farms in Mekelle city, northern Ethiopia. The study followed a controlled before-and-after design, with data collected prospectively at baseline and at three and six months after the intervention. The study area comprised smallholder and semi-intensive commercial dairy operations located within Mekelle city. Mekelle city, the capital city of the Tigray regional state, is located at a distance of 783 km north of Addis Ababa, Ethiopia. The study was conducted from December 2024 to June 2025.

### Farm selection, grouping and participants

No formal a priori sample size calculation was performed, as the study employed a pragmatic design constrained by the number of dairy farms meeting predefined inclusion criteria and willing to participate. Twenty-two dairy farms were purposively selected on the basis of their willingness to participate, record-keeping capability, management practices, and herd size (minimum of 20 lactating cows per farm). The farms were then allocated into intervention (*n* = 11) and control (*n* = 11) groups on the basis of geographic proximity. Geographic allocation was used to minimize environmental and service-related heterogeneity between groups, including climate, feed availability, and access to veterinary care. Purposive selection was applied to ensure inclusion of farms with a minimum herd size, stable management structure, and functional record-keeping systems required for prospective AMU and disease incidence monitoring. The two groups were comparable in herd size, production system, and management practices at baseline. All the participating farms consented to prospective data collection for the duration of the study.

The participants included 68 farm owners and primary farmworkers, 34 from the intervention group and 34 from the control group, directly involved in animal health decision-making and antimicrobial administration. Eligible participants were aged 18 years or older and provided written informed consent before data collection. Each participating farm had at least one respondent who completed the knowledge and attitude questionnaire.

### Intervention description

The AMS training intervention was developed and delivered by the study investigators, with training content partially adapted from on-farm AMS frameworks [21, 24], and emphasized practical skills and behavioral change for responsible AMU. The program consisted of two interactive on-farm sessions delivered in the local language (Tigrigna) two weeks apart and covered modules for fundamentals of AMR and AMS; early recognition of major dairy cattle diseases (mastitis, respiratory disease, metritis, calf diarrhea, lameness); judicious antimicrobial decision-making, including when to treat, when to observe, and when to consult a veterinarian; record-keeping; and prevention and herd health management practices to reduce disease occurrence. The training materials included a pictorial and infographic guide. Attendance was recorded for all sessions, and intervention fidelity was monitored through regular supervisory visits that assessed adherence to the training content and recommended antimicrobial use practices. The control farms received no training during the study period but were offered the same materials at study completion.

### Outcome measurements

#### Knowledge and attitudes toward AMR and AMU

The questionnaire was in line with previously published AMU and stewardship surveys in livestock systems [28, 29] and adapted to the local dairy farming context. The investigators also considered the indicators and guidelines from the World Health Organization (WHO) Global Action Plan on Antimicrobial Resistance (2016) [30], the Food and Agricultural organization (FAO) Action Plan on AMR (2021–2025) [31], and the World Organization for Animal Health (WOAH) Terrestrial Animal Health Code, Chap. 6.10 on the Responsible and Prudent Use of Antimicrobial Agents in Veterinary Medicine (2024) [32]. Item selection was guided by key stewardship domains, including antimicrobial indications, dosing practices, withdrawal periods, and perceptions of AMR. Content validity was assessed through expert review by veterinarians and pharmacologists, and pre-testing was conducted on five non-participating farms to evaluate clarity and relevance. Internal consistency was assessed using Cronbach’s alpha. The knowledge domain demonstrated acceptable reliability (α = 0.78), and the attitude domain demonstrated good reliability (α = 0.82). The English version of the questionnaire is provided as Supplementary File 1.

The knowledge items, a total of 20 questions, assessed participants’ understanding of AMR causes, prudent AMU principles, withdrawal periods, and alternatives to antimicrobials. Each correct response was scored as 1, incorrect or “don’t know” as 0; the total was converted to a percentage score. The attitude items, a total of 10 questions, used a 5-point Likert scale (1 = strongly disagree to 5 = strongly agree) to measure perceptions of responsibility, stewardship willingness, and confidence in applying AMS principles. The mean attitude scores were computed for each participant. The questionnaire was administered to all participants at baseline and at three and six months post-training to the same participants.

#### Antimicrobial use (AMU)

AMU was the primary farm-level outcome and was measured as the number of treatments per 100 cow-days.

$$Treatment\;rate=\frac{Number\;of\;antimicrobial\;treatmet\;events}{Total\;cow\;days\;at\;risk}\times100$$

A treatment event was defined as the initiation of an antimicrobial course for a distinct clinical episode in an individual animal. The number of cow-days at risk was computed from the daily number of cows present on each farm. AMU was recorded prospectively using standardized treatment logs maintained at each farm and reviewed during weekly investigator visits. For each treatment event, data collected included antimicrobial name, class, route of administration, indication, and treatment duration. AMU quantification focused on treatment events rather than dose-based metrics due to variability in dosing practices and record completeness across farms. Intramammary and systemic antimicrobial treatments were recorded separately but aggregated when calculating total AMU expressed as treatments per 100 cow-days. Moreover, repeated antimicrobial administrations for the same disease episode were counted as separate treatment events when administered on different days or as part of a multi-day treatment course, consistent with standard treatment-based AMU metrics. To ensure data accuracy, treatment logs were cross-checked during weekly visits against antimicrobial purchase receipts and inconsistencies were directly clarified with farm personnel.

#### Disease incidence rate

Disease incidence was monitored prospectively through weekly farm visits using predefined operational case definitions. Farm personnel recorded disease events based on observable clinical signs and routine farm records, while trained investigators verified diagnoses during weekly visits to ensure consistency.

To ensure diagnostic consistency, investigators received standardized training on case definitions prior to study initiation. Uniform data collection forms were used across all farms, and reported disease events were cross-checked against clinical observations and treatment records during weekly visits. Any discrepancies were resolved through investigator assessment.

$$Disease\;incidence\;rate=\frac{Number\;of\;new\;disease\;cases}{Total\;cow\;days\;at\;risk}\times100$$

The following case definitions were operationalized and harmonized with the training modules to ensure consistency.

- ▪ Mastitis was defined as the presence of abnormal milk (clots, flakes, watery appearance) with or without visible udder inflammation (swelling, heat, pain), as reported by farm personnel and confirmed during weekly visits.
- ▪ Respiratory disease was defined as the presence of coughing, nasal discharge, increased respiratory rate, or labored breathing.
- ▪ Metritis was defined as the presence of foul-smelling uterine discharge within 21 days postpartum, with or without systemic signs such as fever or reduced appetite.
- ▪ Lameness was defined as visible gait abnormality, with or without a wound, affecting normal locomotion.
- ▪ Calf diarrhea was defined as the passage of loose or watery faces with increased frequency compared to normal.

### Data collection procedures and analysis

Data collection took place over approximately six months. A baseline phase of eight weeks preceded the AMS training, followed by post training monitoring at three and six months. The study investigators conducted weekly farm visits to collect and validate the data. Knowledge and attitude questionnaires were administered face-to-face.

All the data were coded, entered into EpiData and exported to Stata for analysis. Descriptive statistics summarized the baseline characteristics of the farms and participants. Continuous variables are reported as the means ± standard deviations (SDs), and categorical variables as frequencies and percentages. A difference-in-differences (DiD) analytical approach was used to estimate the effect of AMS training on AMU and knowledge and attitude scores. For continuous outcomes (treatment rate and knowledge/attitude scores), linear mixed-effects models with farm-level random intercepts were fitted to account for clustering and repeated measures (extended information is provided in the Supplementary File 2).

Disease incidence rates were analysed via mixed-effects Poisson regression with the natural log of cow days as an offset variable. All models were adjusted for herd size and baseline outcome values. Statistical significance was set at *p* < 0.05.

## Results

### Farm and participant characteristics

The mean herd size was 26.4 ± 3.6 cows for the intervention farms and 25.5 ± 4.2 cows for the control farms. The majority (84%) of the respondents were male, and 68% had completed at least secondary education (Table 1).

**Table 1:** Baseline characteristics of the participating dairy farms and respondents

Characteristic	Intervention (*n* = 11)	Control (*n* = 11)	*p* value
Mean herd size (mean ± SD)	26.4 ± 3.6	25.5 ± 4.2	0.32
Production system (semi-intensive, %)	89%	92%	0.61
Record-keeping practices present (%)	85%	91%	0.59
Mean respondent age (years ± SD)	36.8 ± 7.4	37.5 ± 6.9	0.78
Education level ≥ secondary (%)	67%	69%	0.72
Mean years of dairy experience	7.4 ± 3.2	7.9 ± 3.5	0.68

### Knowledge and attitudes toward AMR and AMU

A total of 68 participants completed the questionnaire at baseline, representing at least one responsible individual per farm. The same participants were followed and re-administered the questionnaire at the midline and endline assessments. At baseline, the knowledge and attitude scores were similar between the two groups. Following the AMS training, the mean knowledge scores in the intervention group changed from 48.2% ± 12.5 at baseline to 79.4% ± 9.8 at three months and 78.6% ± 10.2 at six months. In contrast, the scores of the control group changed from 47.3% ± 11.7 to 51.5% ± 10.9 at six months. The attitudes toward responsible AMU followed a similar trend, changed from a baseline mean of 3.1 ± 0.6 to 4.3 ± 0.4 in the third month and 4.2 ± 0.5 in the sixth month among the intervention group, whereas the scores changed from 3.0 ± 0.5 to 3.2 ± 0.5 in the sixth month among the controls (Table 2).

**Table 2:** Changes in knowledge and attitudes toward AMR and AMU among participants

Outcome	Time point	Intervention (mean ± SD)	Control (mean ± SD)	DiD (95% CI)	*p* value
Knowledge score (%)	Baseline	48.2 ± 12.5	47.3 ± 11.7	-	-
	3 months	79.4 ± 9.8	50.6 ± 10.3	28.8 (20.9, 36.5)	< 0.05
	6 months	78.6 ± 10.2	51.5 ± 10.9	27.1 (20.2, 35.4)	< 0.05
Attitude score (1–5 Likert)	Baseline	3.1 ± 0.6	3.0 ± 0.5	-	-
	3 months	4.3 ± 0.4	3.1 ± 0.5	1.2 (0.7, 1.4)	< 0.05
	6 months	4.2 ± 0.5	3.2 ± 0.5	1.0 (0.6, 1.3)	< 0.05

*DiD* Difference-in-differences

A DiD analysis showed that the AMS intervention may improved knowledge in the three-month period (β = 28.8%, *p* < 0.05) and the six-month period (27.1%, *p* < 0.05). Similarly, the attitude scores at three months (β = +1.2 points, *p* < 0.05) and six months (+ 1.0-point, *p* < 0.05) changed differently compared with those of the controls (Table 2).

### Antimicrobial use (AMU)

A total of 1,136 antimicrobial treatment events were recorded across all farms during the study period. At baseline, the mean antimicrobial treatment rates were 4.5 treatments per 100 cow-days on the intervention farms and 4.2 treatments per 100 cow-days on the control farms. Following the AMS training, treatment rates in the intervention farms changed to 2.1 per 100 cow-days at three months and 2.0 per 100 cow-days at six months, representing a 56% overall reduction from baseline. In contrast, control farms showed only a modest reduction from 4.1 to 3.9 treatments per 100 cow-days over the same period (Table 3).

**Table 3:** Changes in antimicrobial use, treatments per 100 cow-days

Time point	Intervention (mean ± SD)	Control (mean ± SD)	Percent change (intervention)	DiD estimate (95% CI)	*p* value
Baseline	4.5 ± 1.2	4.1 ± 1.0	-	-	-
3 months	2.1 ± 0.9	4.0 ± 1.1	-53%	-2.3 (-3.1, -1.8)	< 0.05
6 months	2.0 ± 0.8	3.9 ± 1.0	-56%	-2.2 (-2.9, -1.6)	< 0.05

Mixed-effects regression showed that AMS training was associated with a reduction in AMU (β = -2.3 treatments/100 cow-days; 95% CI: -3.1, -1.8; *p* < 0.05), with a 53% change in the third month and (β = -2.2 treatments/100 cow-days; 95% CI: -2.9, -1.6); *p* < 0.05), with a 56% reduction in the sixth month. This finding indicates that AMS training could cause a reduction in AMU, with estimated mean decreases of 2.3 and 2.2 treatments per 100 cow-days in the third and sixth months, respectively (Table 3).

### Disease incidence rate

Across all farms, the most common diseases recorded were mastitis, metritis, respiratory disease, and calf diarrhea. Lameness of different causes was also a disease incident. The baseline mean disease incidence was 7.9 cases per 100 cow-days on the intervention farms and 7.2 on the control farms. At six months, the incidence changed to 4.5 cases per 100 cow-days in the intervention group, a 43% reduction from baseline, whereas it remained relatively stable in the control farms (7.3 cases per 100 cow-days). The greatest changes were observed for mastitis (-52%) and respiratory disease (-47%) (Table 4).

**Table 4:** Disease incidence rates by disease category, time period and mixed-effects Poisson regression results

Disease category	Intervention		% Change	Control		IRR	95% CI	*p* value
	Baseline	6 months		Baseline	6 months			
Mastitis	2.7	1.3	-52%	2.4	2.5	0.48	0.35–0.66	0.008
Respiratory disease	1.9	1.0	-47%	1.8	1.7	0.52	0.38–0.72	0.009
Metritis	1.5	0.9	-40%	1.4	1.6	0.58	0.41–0.83	0.020
Calf diarrhea	1.1	0.7	-36%	1.0	1.0	0.63	0.44–0.91	0.030
Lameness	0.7	0.6	-14%	0.6	0.5	0.82	0.60–1.13	0.210
Total	7.9	4.5	-43%	7.2	7.3	0.54	0.42–0.69	< 0.05

*IRR* Incidence rate ratio, *CI* Confidence interval

As shown in Table 4, the mixed-effects Poisson regression analysis showed that the AMS intervention reduced total disease incidence (incidence rate ratio [IRR] = 0.54; 95% CI: 0.42–0.69; *p* < 0.05) after adjusting for herd size and baseline incidence. Disease-specific models further showed a reduction in mastitis (IRR = 0.48; 95% CI: 0.35–0.66; *p* < 0.05), respiratory disease (IRR = 0.52; 95% CI: 0.38–0.72; *p* < 0.05), metritis (IRR = 0.58; 95% CI: 0.41–0.83; *p* < 0.05), and calf diarrhea (IRR = 0.63; 95% CI: 0.44–0.91; *p* < 0.05). Lameness showed a smaller decline (IRR = 0.82; 95% CI: 0.60–1.13; *p* = 0.21). The model demonstrated good fit (AIC = 1027.4), and farm-level random effects accounted for 12.3% of the variance.

## Discussion

The present study demonstrated improvement in participants’ knowledge and attitudes following the AMS training intervention. The knowledge scores of the intervention farms changed from 48.2% at baseline to 79.4% and 78.6% at three and six months, respectively, whereas the attitude scores changed from 3.1 to 4.3 and 4.2 during the same period, compared with the minimal change in the control group. These findings align with several reports indicating that targeted stewardship training may promote awareness and perceptions of AMR among livestock stakeholders. For example, Portillo-Gonzalez et al. [20]. and Garzon et al. [21]. demonstrated that on-farm AMS education improved farmers’ understanding of prudent AMU and their willingness to adopt responsible practices. Additionally, these findings are consistent with previous AMS intervention studies on livestock farms in other countries, which have shown that participatory and context-based training can shift farmers’ understanding and perceptions of AMR [24].

Although there are no reported studies conducted in Eastern Africa on the effects of AMS intervention on dairy farms, the baseline assessment indicates poor knowledge and unfavourable attitude towards AMR and AMU. A study conducted by Kallu et al. revealed that only 57.7% of dairy farm owners/workers in Addis Ababa had good knowledge of AMU and AMR and that fewer than half (47.7%) held desirable attitudes toward AMU and AMR [29]. Similarly, a recent livestock producers study in the Buno Bedele and Ilu-Babor zones of Ethiopia revealed that 94% of farmers lacked adequate knowledge and that 97% had negative attitudes (< 50% correct answers) [33]. In a study conducted in Rwanda among cattle farmers, only about 52.6% had correct knowledge and 56% had favorable attitudes toward AMR and AMU [34]. Compared with these findings, the postintervention knowledge (79%) and attitude (4.2/5) levels observed in the sixth month of this study may indicate the effects of AMS training at the farm level.

The changes observed here may be attributed to several factors. First, the training was delivered onsite, in the local Tigrigna language, and was reinforced with infographic guides and practical farm scenarios, which may have facilitated better comprehension and retention than conventional lecture-based approaches. Second, the interactive two-session training delivered over two weeks, combined with subsequent follow-up and outcome assessments at three and six months, may have contributed to knowledge retention and positive behavioral reinforcement. Additionally, the participants in this study, primarily farm owners and workers directly involved in disease treatment decisions, were strongly motivated to integrate new knowledge into their daily work. The relatively low baseline knowledge and attitude scores may have also allowed for greater observable improvement following the intervention, as reported by other studies that farmers without any previous AMS training had increased knowledge scores [35, 36]. Farmers’ attitudes towards good practices of AMU may be familiar, to a certain degree, with general terms and concepts related to AMS [37, 38], but there is a need to improve dairy farmers’ practices in basic diagnostics and treatment to achieve more prudent use of antimicrobials [39]. In addition to this, focusing on the poorly understood items of knowledge and attitude of farmers towards AMU and AMR is recommendable as the current study focused on overall knowledge and attitude scores to evaluate changes over time.

On the other hand, the magnitude of AMU reduction in this study (56%) is consistent with results from other on-farm stewardship evaluations, although reported effect sizes vary by context and intervention design. Pempek et al.. reported an approximately 50% reduction in the dosing rate among calf producers after AMS training and benchmarking intervention, demonstrating that targeted education plus feedback may halve antimicrobial initiation rates on farms [24]. Similarly, a study conducted by Lipkens et al.. reported an average 22% reduction in udder health-related AMU with selective dry-cow policies, indicating that both behavior-focused training and technical changes can reduce use but to differing extents depending on the target and scope [40]. Another recent quasi experimental work from United States dairy systems also reveals the beneficial effects of combined training and benchmarking on farm AMU, further supporting the plausibility of the reductions we observed [41].

Several mechanisms may explain the decline in the treatments observed in this study. First, baseline AMU on participating farms was relatively high, creating greater potential for rapid reductions following AMS; however the training emphasized diagnose-before-treat principle, which reduces impulsive initiation of antimicrobial courses; similar diagnose-before-treat effects have been described in other stewardship trials [21]. Second, the promotion of prevention and herd health measures (hygiene, milking best practices, colostrum/calf care, and prompt isolation of sick animals) may lowered the true disease incidence and therefore treatment demand, although the effect of season on disease occurrence and calving was not controlled. Third, improved record-keeping and weekly monitoring may create accountability and makes unnecessary or repeat courses easier to detect and avoid; feedback and benchmarking approaches have been shown to motivate reductions in use in farm settings [41]. These combined pathways, preventive improvements, and record-based accountability align with the stewardship elements recommended in a recent review of on-farm AMS and practical experiences [42].

In this study, the overall disease incidence ranged from 7.9 to 4.5 cases per 100 cow-days on the intervention farms (43% reduction), with the relative decreases observed for mastitis (52%) and respiratory disease (47%). The mixed-effects Poisson regression also showed that AMS training was associated with reduced overall disease incidence: the adjusted incidence rate ratio (IRR) was 0.54 (95% CI: 0.42–0.69; *p* < 0.05), indicating a 46% lower rate of new disease cases in intervention farms than in controls. Such reductions are may be caused due to focused herd health and stewardship interventions in dairy systems. National mastitis control programmes and targeted udder-health interventions have produced clinically meaningful declines in mastitis incidence; for example, a coordinated mastitis control intervention in England and Wales produced an approximate 22% reduction in cows affected by clinical mastitis after one year [43]. More targeted on-farm management strategies, including improved milking hygiene, prompt case detection, and selective dry-cow therapy, have also been associated with reductions in udder-health problems; intervention studies and reviews report reductions ranging from approximately 20% to larger declines depending on the baseline incidence and completeness of implementation [44]. The effect of this study’s intervention is also compatible with stewardship programs that combine training with prevention. Garzon et al.. on-farm training framework emphasized disease recognition and protocol adherence and documented improved case recognition and changes in worker behavior that are known precursors to reduce disease burden [21]. Another study in the United States reported that structured AMS programs leveraging disease prevention and early detection can lead to measurable declines in disease incidence [42]. These results support the use of farm-level AMS training as part of integrated herd health programs in low-resource dairy systems while indicating the need for longer-term follow-up and microbiological surveillance to confirm downstream impacts on AMR.

There may be several plausible mechanisms linking AMS training to lower disease incidence in this study. The training focus on early identification of clinical signs and proper triage (treat, observe, escalate), may led to earlier nonantimicrobial interventions (isolation, supportive care, improved hygiene). Additionally, prevention modules that directly target drivers of mastitis and respiratory disease may cause a great effect; as the systematic application measures has been shown repeatedly to reduce disease incidence in other settings [45]. The prospective weekly monitoring and improved record-keeping that were part of the study’s protocol may have improved case follow-up and corrective management, an effect similar to that reported in other stewardship intervention studies where monitoring and feedback have been central to improvements [24]. On the other hand, the observed reductions may partially reflect changes in measurement and reporting rather than purely biological effects. Improved record-keeping following training and closer engagement with study personnel may have influenced how treatments and disease events were documented. An observer (Hawthorne) effect, whereby farmers modify behavior due to awareness of being monitored, may also have contributed to the observed declines, particularly during the early post-intervention period.

The current study demonstrated that AMS training in dairy farms was associated with positive change of farmers’ knowledge and attitudes toward responsible AMU and linked with reductions in both AMU and disease incidence rates. These findings align with evidence from similar interventions conducted in other livestock systems worldwide, where participatory and context-based training approaches have produced behavioral change [21, 24]. The continued improvements observed between the three- and six-month may suggest that the effects of the intervention may have extended beyond the immediate training period. As farmers gained practical experience applying stewardship principles, informal learning processes, such as discussions on AMU with peers and veterinarians, may have contributed to sustained improvement. These findings also suggest that AMS interventions may act as catalysts rather than isolated events. While the study design does not allow attribution of these changes to specific post-training influences, the observed effects supports the potential for sustained impacts of stewardship training in smallholder dairy systems.

From a One Health perspective, such interventions are crucial because they address AMR at its agricultural origin, before resistant bacteria and genes can circulate through food, environment, and occupational pathways to human populations [10, 46]. Ethiopian studies have shown that resistant pathogens such as *Escherichia coli* and *Campylobacter* are frequently shared between livestock, farm workers, and surrounding environments [47, 48]. Therefore, reducing antibiotic exposure pressure and promoting better infection prevention on farms, AMS programs directly contribute to breaking these transmission cycles and safeguards antimicrobial effectiveness for both veterinary and human medicine. While knowledge and attitude gains are promising, sustained behaviour change requires ongoing veterinary support, monitoring, and enabling policy frameworks, because these mechanisms are particularly significant in low-resource dairy systems [49].

### Limitations of the study

This study has several limitations that should be considered when interpreting the findings. Seasonal variations may have influenced the observed outcomes; subtle differences in herd management, access to veterinary services, or biosecurity could not be entirely controlled for. While the AMS training focused on practical skills and behavioral change, the relatively short follow-up period limits the assessment of the long-term sustainability of improved knowledge and attitudes and reduced AMU. Additionally, microbiological outcomes such as AMR patterns in bacterial isolates were not included; therefore, we cannot directly link reduced AMU and disease incidence with changes in resistance levels on the farms. The use of purposive farm selection and non-random geographic allocation may have introduced selection and contamination bias. The relatively small number of farms and respondents may limit statistical power, particularly for disease-specific incidence outcomes and subgroup analyses. However, the study should be considered exploratory with respect to individual disease categories. Although internal consistency was acceptable, the questionnaire was not subjected to formal construct validation or factor analysis due to sample size constraints and the applied nature of the study. A limitation of the DiD analysis is the absence of multiple pre-intervention observations to formally test the parallel trends assumption. Despite these limitations, longitudinal, prospective data collection provides preliminary evidence that farm-level AMS training can improve stewardship practices and herd health outcomes in resource-limited dairy systems.

## Conclusion

This field-based intervention demonstrated that a structured AMS training program may improve the knowledge and attitudes of dairy farm workers toward prudent AMU while reducing antimicrobial consumption and disease incidence on farms. The intervention resulted in an approximate 30% increase in knowledge scores, a one-point increase in attitude scores, a 56% reduction in antimicrobial treatment rates, and a 43% decrease in overall disease incidence within six months. These findings could be good indication that participatory, context-specific AMS training is a practical and effective strategy for improving farm management, minimizing unnecessary AMU, and enhancing animal health outcomes. Integrating AMS training into routine veterinary extension services and dairy herd management programs could achieve sustainable improvements in AMU and contribute to slowing the emergence and spread of AMR, with potential benefits for both animal and human health. Future research should evaluate the long-term retention of these behavioral changes, incorporate microbiological monitoring to link farm-level practices with resistance trends, and explore scalability across diverse production systems in Ethiopia and comparable settings.

## Supplementary Information

Supplementary Material 1.

Supplementary Material 2.

## Data Availability

The data used and/or analysed during the study are available from the corresponding author upon reasonable request.

## Abbreviations

AMR: antimicrobial resistance
AMS: antimicrobial stewardship
AMU: antimicrobial use
DiD: Difference-in-differences
IRR: Incidence rate ratio
