# Supplementary material for: Promoting antimicrobial stewardship on dairy farms in Mekelle, Northern Ethiopia: a field intervention with One Health implications
Source: BMC Vet Res. 2026 Mar 9;22:233. doi: 10.1186/s12917-026-05385-z (PMC13085486; doi:10.1186/s12917-026-05385-z)
Supplement: Supplementary file 1 — Supplementary Material 1. [file 12917_2026_5385_MOESM1_ESM.docx]

**Supplementary File 1. Farmer Questionnaire (English Version)**

**Title**: Knowledge and Attitudes toward Antimicrobial Use and Antimicrobial Resistance among Dairy Farmers in Mekelle, Northern Ethiopia

**Study**: **Promoting Antimicrobial Stewardship on Dairy Farms in Mekelle, Northern Ethiopia: A Field Intervention with One Health Implications**

**Purpose**: This questionnaire was developed specifically for the above-mentioned study to assess changes in farmers’ knowledge and attitudes regarding antimicrobial use (AMU) and antimicrobial resistance (AMR) before and after the antimicrobial stewardship (AMS) training intervention.

**Instructions**: Please read each question carefully and choose the best answer. All responses will remain confidential and will be used only for research purposes.

**Section 1. Demographic and Farm Characteristics**

1. Age of respondent: _____ years
2. Sex: ☐ Male ☐ Female
3. Educational level: ☐ No formal education ☐ Primary ☐ Secondary ☐ Diploma ☐ Degree or higher
4. Years of dairy farming experience: _____ years
5. Number of lactating cows currently on the farm: _____
6. Do you have regular access to veterinary services? ☐ Yes ☐ No
7. Who usually decides when and how to use antibiotics on your farm?
   ☐ Veterinarian ☐ Farm owner/manager ☐ Worker ☐ Drug seller
8. Have you received any formal training on antimicrobial use before this study? ☐ Yes ☐ No

**Section 2. Knowledge about Antimicrobial Use and Antimicrobial Resistance**

(Tick one response for each question)

| **SN** | **Question** | **Response options** |
| --- | --- | --- |
| 1 | Antibiotics are medicines used to treat bacterial infections. | ☐ True ☐ False ☐ Not sure |
| 2 | Antibiotics can cure viral infections like lumpy skin disease. | ☐ True ☐ False ☐ Not sure |
| 3 | Overusing antibiotics can make them less effective in the future. | ☐ True ☐ False ☐ Not sure |
| 4 | Stopping antibiotic treatment too early can lead to resistance. | ☐ True ☐ False ☐ Not sure |
| 5 | Each antibiotic should be used only for the specific infection it was prescribed for. | ☐ True ☐ False ☐ Not sure |
| 6 | Withdrawal periods must be respected before milk from treated cows is sold. | ☐ True ☐ False ☐ Not sure |
| 7 | Poor housing and hygiene increase the need for antibiotics. | ☐ True ☐ False ☐ Not sure |
| 8 | The same antibiotic can be used to treat all diseases. | ☐ True ☐ False ☐ Not sure |
| 9 | Antibiotic resistance occurs when bacteria are no longer killed by antibiotics. | ☐ True ☐ False ☐ Not sure |
| 10 | Antibiotic resistance can spread from animals to animals and from animals to humans. | ☐ True ☐ False ☐ Not sure |
| 11 | A veterinarian should always be consulted before antibiotics are used. | ☐ True ☐ False ☐ Not sure |
| 12 | Cleaning and disinfecting barns can reduce the need for antibiotics. | ☐ True ☐ False ☐ Not sure |
| 13 | Using leftover antibiotics is acceptable if the animal shows similar symptoms. | ☐ True ☐ False ☐ Not sure |
| 14 | Recording animal treatments helps prevent unnecessary antibiotic use. | ☐ True ☐ False ☐ Not sure |
| 15 | Antimicrobial resistance is a growing problem in dairy production. | ☐ True ☐ False ☐ Not sure |
| 16 | Infections caused by resistant bacteria are harder and more expensive to treat. | ☐ True ☐ False ☐ Not sure |
| 17 | Using antibiotics as a preventive measure without disease signs is acceptable. | ☐ True ☐ False ☐ Not sure |
| 18 | Antibiotic residues in milk can be harmful to consumers. | ☐ True ☐ False ☐ Not sure |
| 19 | Following the prescribed dose and duration helps reduce AMR. | ☐ True ☐ False ☐ Not sure |
| 20 | Farmworkers can contribute to reducing antimicrobial resistance. | ☐ True ☐ False ☐ Not sure |

**Section 3: Attitudes toward Antimicrobial Use and Resistance**

(Please indicate your level of agreement with each statement)

Scale: 1 = Strongly disagree, 2 = Disagree, 3 = Neutral, 4 = Agree, 5 = Strongly agree

| **No** | **Statement** | **1** | **2** | **3** | **4** | **5** |
| --- | --- | --- | --- | --- | --- | --- |
| 1 | Responsible use of antibiotics is important to protect animal health. | ☐ | ☐ | ☐ | ☐ | ☐ |
| 2 | Veterinary guidance should always be sought before using antibiotics. | ☐ | ☐ | ☐ | ☐ | ☐ |
| 3 | Reducing antibiotic use will lower milk production. | ☐ | ☐ | ☐ | ☐ | ☐ |
| 4 | Hygiene and good management can reduce the need for antibiotics. | ☐ | ☐ | ☐ | ☐ | ☐ |
| 5 | Antibiotic resistance is a serious problem on dairy farms. | ☐ | ☐ | ☐ | ☐ | ☐ |
| 6 | Keeping treatment records is unnecessary if the farmer remembers what was given. | ☐ | ☐ | ☐ | ☐ | ☐ |
| 7 | Following withdrawal periods is important to ensure milk safety. | ☐ | ☐ | ☐ | ☐ | ☐ |
| 8 | Antibiotics should only be used when prescribed by a veterinarian. | ☐ | ☐ | ☐ | ☐ | ☐ |
| 9 | Farmers can play a role in preventing antimicrobial resistance. | ☐ | ☐ | ☐ | ☐ | ☐ |
| 10 | It is acceptable to share leftover antibiotics between animals. | ☐ | ☐ | ☐ | ☐ | ☐ |

Data collectors name: _________________________________ Signature: _________________
