# Supplementary material for: Promoting antimicrobial stewardship on dairy farms in Mekelle, Northern Ethiopia: a field intervention with One Health implications
Source: BMC Vet Res. 2026 Mar 9;22:233. doi: 10.1186/s12917-026-05385-z (PMC13085486; doi:10.1186/s12917-026-05385-z)
Supplement: Supplementary file 2 — Supplementary Material 2. [file 12917_2026_5385_MOESM2_ESM.docx]

**Mixed-effects model specifications and diagnostics**

1. **Statistical modelling framework**

To account for repeated measurements within farms and clustering at the farm level, mixed-effects regression models were used to analyse antimicrobial use (AMU) and disease incidence outcomes over time. The primary analytical approach followed a difference-in-differences (DiD) framework implemented within a mixed-effects model structure.

1. **Model specification**
   1. **Fixed effects**

For each outcome, the following fixed effects were included:

- Intervention group (Intervention vs control)
- Time period (Pre-intervention vs post-intervention)
- Interaction term between intervention group and time period

The interaction term represents the difference-in-differences estimator, capturing the intervention-associated change beyond temporal trends common to both groups.

Random effects: Random slopes were not included due to the limited number of clusters and convergence considerations.

1. **Model equations:** The general mixed-effects DiD model was specified as:

*Y*_it_ = *β*_0_ + *β*_1_ Intervention*_i_* + *β*_2_ Post*_t_* + *β*_3_ (Intervention*_i_* × Post*_t_*) + u*_i_* + ε*_it_*

Where:

- *Y*_it_ = outcome for farm *i* at time *t* (antimicrobial use expressed as treatments per 100 cow-days, or disease incidence)
- *β*_0_​ = overall intercept
- *β*_1_​ = baseline difference between intervention and control farms
- *β*_2_​ = common temporal effect
- *β*_3_​ = difference-in-differences estimator (intervention effect)
- u*_i_* ​ = random intercept for farm *i*
- ε*_it_*  = residual error term

1. **Intraclass correlation coefficients (ICCs)**

Intraclass correlation coefficients (ICCs) were calculated from unconditional (null) mixed-effects models containing only the random intercept for farm. ICCs quantify the proportion of total variance attributable to between-farm clustering.

- Antimicrobial use outcome: ICC = 0.21
- Disease incidence outcome: ICC = 0.18

1. **Model diagnostics**

Model assumptions were evaluated using standard diagnostic procedures:

- Residual vs fitted value plots were inspected to assess homoscedasticity.
- Quantile-quantile (Q-Q) plots of residuals were examined to assess normality.
- No major deviations from model assumptions were observed.

All models demonstrated acceptable convergence and stability.

1. **Interpretation note**

Given the pragmatic, field-based nature of the study and the limited number of clusters, models were specified to balance statistical rigor with feasibility. The mixed-effects DiD approach allows estimation of intervention-associated changes while accounting for clustering and repeated measures, but results should be interpreted as indicative of real-world effectiveness rather than definitive causal effects.
